# Intra-Rater Test–Retest Reliability of Static Dual-Task Balance Measures in Untrained People with Parkinson’s Disease

**DOI:** 10.3390/neurolint18100187

**Published:** 2026-10-01

**Authors:** Sara Monleón Guinot, Constanza San Martín Valenzuela, Vivina Aranda Asensi, José M. Tomás

**Affiliations:** 1 Department of Methodology for the Behavioral Sciences, University of Valencia, 21 Blasco Ibáñez Av., 46010 Valencia, Spain; 2 Department of Physiotherapy, University of Valencia, 5 Gascó Oliag St., 46010 Valencia, Spain; 3 Unit of Personal Autonomy, Dependency and Mental Disorders Assessment, University of Valencia-INCLIVA Biomedical Research Institute, 15 Blasco Ibáñez Av., 46010 Valencia, Spain; 4 Centro de Investigación Biomédica en Red de Salud Mental (CIBERSAM), Instituto de Salud Carlos III, 3-5 Monforte de Lemos St., 28029 Madrid, Spain; 5 Physiotherapy Unit, Parkinson Valencia Neurorehabilitation Center, 15 Nicolau Primitiu St., 46014 Valencia, Spain

**Keywords:** balance, dual-task, intra-rater test–retest, Parkinson’s disease, reliability

## Abstract

**Background/Objectives**: Dual-task (DT) motor assessment in Parkinson’s disease (PD) provides insight into functional abilities, yet limited evidence on its reliability may bias therapy results. This study aims to evaluate the intra-rater test–retest reliability of single and functional DT balance measures in untrained individuals with PD, and to determine the Standard Error of Measurement (SEM) and the Minimal Detectable Change (MDC). **Methods**: In this cross-sectional study, 22 untrained participants with PD performed a balance assessment twice, 30 min apart. The Romberg test was measured on a force platform under five conditions: single-task, two cognitive secondary tasks (visual, DT_visual_; verbal, DT_verbal_) and two manual tasks (below 90° of shoulder flexion, DT_lower_; above 90°, DT_upper_). Test–retest reliability of Center of Pressure (CoP) outcomes was assessed using the Intraclass Correlation Coefficient (ICC) besides SEM and MDC. **Results**: Displacement, dispersion, force, swept area, and velocity of CoP movements showed excellent reliability (ICC > 0.75) under the single-task condition, except for anteroposterior displacement, which showed an ICC of 0.73. Swept area (ICC = 0.92), followed by CoP velocity (ICC = 0.90) and anteroposterior CoP force (ICC = 0.90), exhibited the highest reliability. Under dual-task conditions, reliability was more variable across outcomes and task types. CoP velocity showed the most consistent reliability (ICC = 0.74–0.92), whereas swept area showed lower reliability in all DT conditions, but DT_upper_ (ICC = 0.90). Mediolateral CoP outcomes were more reliable than anteroposterior displacement and dispersion, while anteroposterior CoP force outperformed mediolateral force under DT conditions. **Conclusions**: Single-task static balance can be reliably assessed in untrained people with mild-to-moderate PD within a single session. However, under DT conditions, high variability across outcomes warrants cautious interpretation, highlighting CoP velocity and mediolateral excursion as relatively consistent outcomes.

## 1. Introduction

There is growing interest in reproducing challenging environments in physical rehabilitation due to their advantages for motor learning in Parkinson’s disease (PD) [1,2]. Simulations of daily-life conditions are made possible by incorporating dual-task (DT) [3] into physiotherapy, which also introduces a more realistic assessment of motor performance. Differences between single and DT performance in PD have been demonstrated in gait [4] and balance [5,6]. However, DT assessment reliability may be compromised, particularly in neurological disorders where motor variability is common [7,8,9]. In PD, motor fluctuations that are not attributable to controlled factors may arise naturally as a result of disease progression and pharmacological effects [10]. Therefore, studies reporting intervention effects should consider whether observed changes exceed inherent performance variations. Such variability can be examined through test–retest analysis, including the Minimal Detectable Change (MDC), which distinguishes true change from measurement error [11].

The reproducibility of DT assessment in PD has been previously studied in walking [11,12], showing moderate to excellent reliability for gait parameters. In contrast, few studies have addressed balance consistency under single- or dual-task conditions. Previous authors have focused on non-instrumented tests, such as the Tinetti Mobility Test [13], Berg Balance Scale [14,15], Balance Evaluation Systems Test [15], Timed Up and Go Test [14], forward and backward functional reach [14], and patient-perceived outcomes such as the Activities Specific Balance Confidence Scale [14]. Alternatively, the reliability of instrumented assessment with a force platform to analyze Center of Pressure (CoP) parameters is scarce in PD. Two studies reported excellent intra-rater reliability in the single-task condition for ellipse area, velocity, sway amplitude, and CoP frequency during the Romberg test with eyes open [16], and through derived scores from static and dynamic balance assessments on the force platform [17]. Likewise, the MDC values reported for single-task balance outcomes were small, ranging from 0 cm for CoP sway to 1.8 cm^2^ for CoP ellipse area [16]. Conversely, Terra et al. [16] reported poor to moderate reliability under DT conditions in the tandem stance, when participants performed secondary arithmetic tasks.

Beyond the limited literature on DT balance reliability in PD, methodological discrepancies should be considered. Previous studies included participants with mild to moderate disease severity (Hoehn & Yahr scale: 2.3–2.6), who generally maintained stable balance. Furthermore, cognitive status and non-pharmacological therapy participation were often unreported [16,17], despite their potential impact on balance control and measurement repeatability. In light of these gaps, this study aimed to evaluate the intra-rater test–retest reliability of these functional DT balance measures in untrained PD patients [18], and to determine both the Standard Error of Measurement (SEM) and MDC for static balance under single- and dual-task conditions. In this study, functional DT combines postural control with a cognitive or motor task designed to reflect situations commonly encountered in daily life.

## 2. Materials and Methods

### 2.1. Study Design and Setting

This work was a single-day, intra-rater test–retest reliability study based on a secondary analysis of baseline data from the balanceHOME randomized controlled trial [18] (NCT04963894). The manuscript follows the STROBE Statement [19] and COSMIN standards [20]. It was approved by the University of Valencia Human Research Ethics Committee (No. 1686831; 3 July 2021) in accordance with the Declaration of Helsinki and the Council of Europe Convention regarding human rights. Recruitment occurred at the Parkinson Valencia Neurorehabilitation Center, while informed consent signing, eligibility, and reliability assessments were conducted at the University of Valencia. During the first phase (August–September 2021), participants completed the baseline assessment twice with the same rater to determine test–retest reliability.

### 2.2. Participants

Participants under regular neurology care were recruited via the rehabilitation center, with outreach targeting those not engaged in physical therapy, exercise programs, or regular sports. The inclusion criteria were: (1) having idiopathic PD with no atypical features; (2) having stable PD medication for ≥1 month; (3) being classified in stages I-IV of the Hoehn & Yahr scale; and (4) being able to walk independently without assistance or assistive devices both indoors and outdoors. The exclusion criteria included: (1) having comorbidities affecting balance or walking; (2) having other neurological conditions; (3) having uncontrolled chronic diseases, including visual, auditory, or metabolic impairments; and (4) having participated in physiotherapy or regular sports during the previous month.

### 2.3. Measurement Procedures and Outcomes

Participants were assessed on a single day during their ON-medication phase. Assessments were scheduled to coincide with the expected therapeutic effect of their usual antiparkinsonian medication, which was confirmed before testing. After providing written informed consent, participants completed a 5-min eligibility screening and a 12-min balance protocol. To avoid fluctuations in Parkinsonian symptoms, test and retest sessions were separated by a 30-min seated rest, during which were collected the following: (1) standardized clinical interview [18]; (2) weight, height, and body mass index were measured with a wall stadiometer and a TANITA SC-240MA scale (Tanita Corporation, Tokyo, Japan); (3) severity of PD was assessed with the Unified Parkinson’s Rating Scale [21] and the Hoehn & Yahr scale [22]; and (4) cognitive status was registered through the Mini-Mental Parkinson Test [23], the SCOPA cognition test [24,25] and the Frontal Assessment Battery [26].

Static balance was assessed using a force platform flush-mounted to the floor (Dinascan/IBV, Biomechanics Institute of Valencia, Valencia, Spain) and the NedSVE^®^/IBV software (version 5.3.0.1, Biomechanics Institute of Valencia, Valencia, Spain) [27], both referenced for neurological [28,29,30,31] and musculoskeletal [32] conditions, and the healthy population [33,34,35]. Balance tests were conducted in a quiet, isolated laboratory to minimize participant distraction. A single evaluator with over 10 years of experience with this equipment performed all assessments.

**Figure 1:**
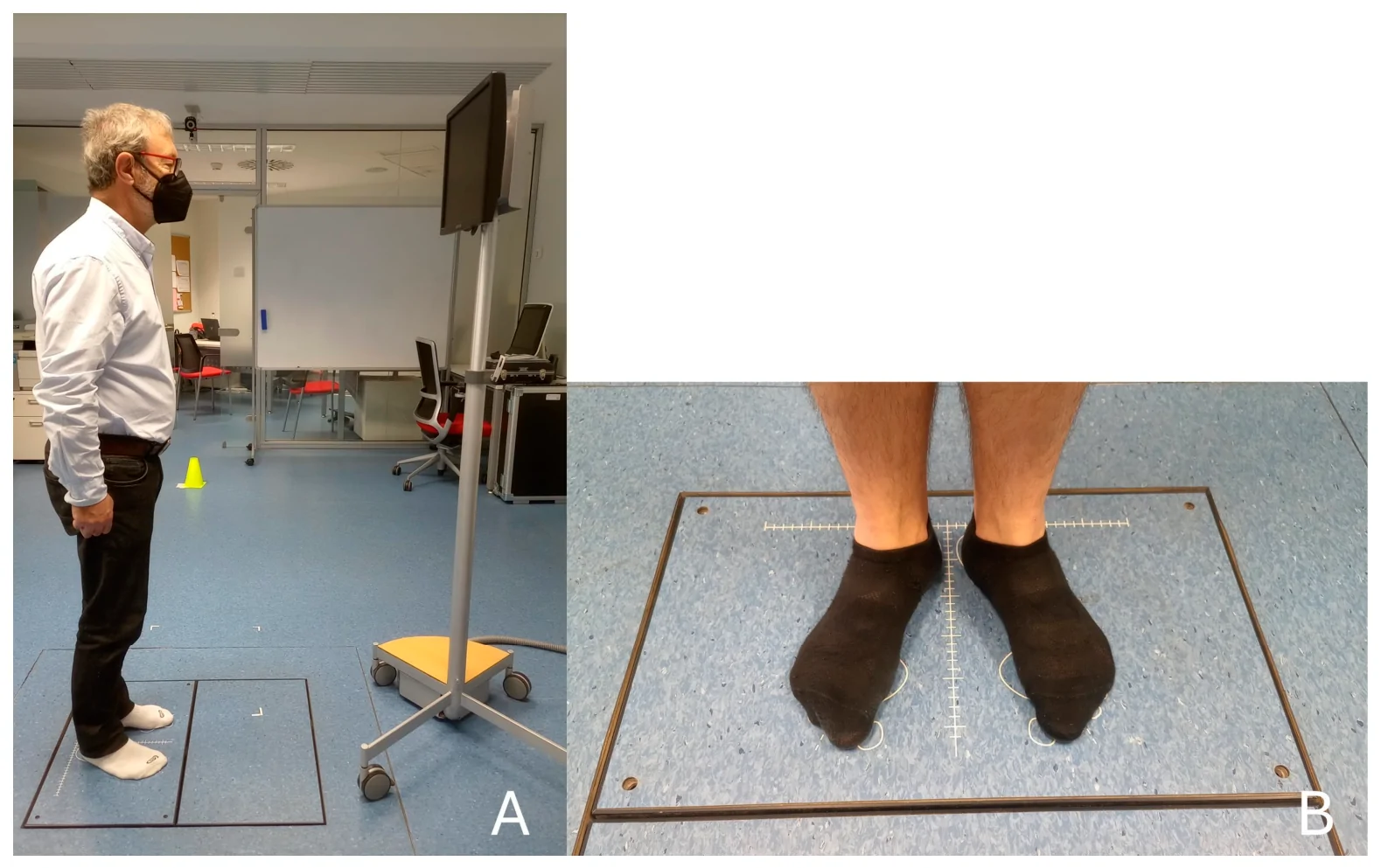
Romberg test position. (**A**) Standing posture during the balance trials. (**B**) Position of the feet on the dynamometric platform, guided by the footprint template.

Participants completed the 30-s Romberg test under five conditions with eyes open and no proprioceptive disturbances. Stance was standardized by remaining barefoot with the arms relaxed at the sides, unless the measurement condition required otherwise (Figure 1A), and feet positioned on a template with toes angled 30° outward and heels 1 cm apart (Figure 1B). Participants maintained a comfortable upright posture, with head orientation fixed on an eye-level screen. They were instructed to remain still, breathe normally, and avoid speaking. Any interruption (e.g., sighing or coughing) terminated the recording, and the trial was repeated to prevent artifactual CoP movements.

The five assessment conditions included one single-task and four DTs simulating daily activities performed in a standing position [4,36]. First, subjects performed the single-task Romberg test, concentrating on standing as stably as possible. Then, the Romberg tests were performed concurrently with a secondary task in a randomized order to prevent learning or fatigue effects. (1) Visual DT (DT_visual_) consisted of viewing successive clock images on a screen, silently focusing on the times shown; afterward, they were asked which times appeared at the beginning and end to verify that they had attended to the secondary task. (2) Verbal DT (DT_verbal_) involved reciting an improvised list of words belonging to a common semantic category. Different categories were used across trials to minimize learning effects while maintaining task fluency. If head movement or speech fluency changed during the test, the trial was repeated to ensure standardization. Secondary motor tasks assessed stability during manual actions below and above shoulder height. (3) In the Motor DT performed with the arms positioned below 90° of shoulder flexion (DT_lower_), participants transferred small objects (such as dry seeds) between two adjacent plastic cups at waist height at their preferred comfortable pace. Lastly, (4) Motor DT with arms raised above 90° shoulder flexion (DT_upper_) comprised simulating the action of brushing one’s hair while maintaining a standardized head position. Participants were instructed to “Hold the comb with one hand and pretend to brush your hair back and forth without touching your head, alternating between the right and left sides. The free hand should accompany the movement of the other.” Only the arms were allowed to move while the head and trunk remained still. To aid understanding, the evaluator demonstrated the test. If visible deviations from the instructed arm movements occurred in either motor task, the trial was stopped and corrected to minimize variability. For each condition, two repetitions of 30 s each were recorded [16], with 30 s of rest between repetitions. The data from these two repetitions were averaged for Test 1 and Test 2 before the statistical analysis. Between measurement conditions, participants rested for one minute [16] in a sitting position.

From each Romberg test, the CoP excursion was studied. Outcomes analyzed were calculated from the mean between repetitions of the same condition, yielding the following: (1) mediolateral and anteroposterior CoP displacement, representing the maximum distance in these directions during exercise execution (mm); (2) CoP swept area, reflecting the area in which participant swaying occurred (mm^2^); (3) CoP velocity, indicating the mean velocity of the participant’s CoP projection during the test (cm/s); (4) mediolateral and anteroposterior CoP dispersion, representing the dispersion of the displacement vector described by the CoP projection in these directions (mm); and (5) mediolateral and anteroposterior force, indicating the maximum force in these directions exerted during the exercise (N) [37,38]. Previous studies have demonstrated that CoP outcomes are sufficiently discriminative to identify alterations in postural patterns in people with PD [39,40]. On the other hand, the primary outcome of the study was the CoP sway area, which is a valid indicator for detecting changes in postural control under dual-task conditions [37,38].

### 2.4. Bias and Sample Size

Sample size was estimated following the recommendation of Borg et al. [41] using the estimation approach and the framework proposed by Bonett et al. (2002) [42] for reliability studies. According to previous literature in PD [16], an expected ICC of 0.83 (0.54–0.93) was assumed for the primary outcome during single-task Romberg test evaluation. To achieve a 95% confidence interval width no greater than 0.30 with *k* = 2 repeated measures, a minimum sample size of 22 participants was required. Dropouts were not considered since both tests occurred during a single appointment.

Potential bias could arise from secondary task complexity and individual patient characteristics, introducing measurement variability. To mitigate this, secondary task performance was standardized, and both evaluations were conducted on the same day with a 30-min interval. This timeframe was selected to minimize potential variability related to medication effects [43] and circadian fluctuations in stress, fatigue, anxiety [44], and sleep quality [45].

### 2.5. Data Analysis

Data were analyzed using IBM SPSS Statistics for Windows version 26 (SPSS Inc., Chicago, IL, USA). For the descriptive variables of the sample, the mean, standard deviation, median, and range were reported. The normality of the distribution of CoP outcomes was assessed with the Shapiro–Wilk test. Normally distributed variables were reported as mean and standard deviation, and differences between Test 1 and Test 2 were evaluated using paired-sample *t*-tests. Conversely, non-normally distributed variables were reported as median and interquartile range, and differences between Test 1 and Test 2 were analyzed using the Wilcoxon signed-rank test. For each paired comparison, mean differences were reported if both repetitions were normally distributed; if either repetition showed a non-normal distribution, median differences were used instead, along with their corresponding *p*-values.

To assess test–retest reliability, single-measure ICC values were calculated using a two-way random-effects model with absolute agreement [11], corresponding to the ICC(2,1) model [46]. This model was selected to allow the generalization of our reliability findings to other clinicians and assessors with similar characteristics [47]. The Shrout and Fleiss’ cutoffs were applied to discriminate the relevance of ICC values: coefficients < 0.40 indicated poor reliability, values between 0.40 and 0.75 reflected fair-to-good reliability, and values > 0.75 suggested excellent reliability [11,16,47]. In addition to ICC, the standard error of measurement (SEM) was estimated to provide an absolute index of reliability to quantify measurement precision in the same units as the measured outcome [48]. The SEM formula used was: $\mathrm{SEM}=S_{\mathrm{pooled}}\times \;\sqrt{\left[1-\mathrm{ICC}\right]}$ [11,48]. Also, the MDC [11,49] was calculated to determine the smallest change in score that can be interpreted as a real difference, beyond the margin of measurement error [49]. We used the formula $\mathrm{MDC}=\mathrm{SEM}\;\times \;1.96\sqrt{2}$ [11,49,50]. Finally, the Bland and Altman plots [11,16,51] were used to visualize the mean difference between pairs of tests registered for the primary outcome, swept area.

## 3. Results

Twenty-two people with PD participated in this study, of whom 63.6% were male and 36.4% female. Their characteristics are presented in Table 1. Regarding the severity of PD, 40.9% of participants exhibited H&Y stage 3, which involves bilateral signs and postural instability. In stage 2, 36.4% of the participants were included, while 22.7% were in stage 1, indicating preserved postural stability during the pull test. Additionally, five participants reported experiencing at least one fall within the year preceding the assessment. Regarding cognitive performance, 25% of the sample scored below 25 on the MMP examination, indicating cognitive impairment [52,53]. Similarly, 33.3% of the participants obtained a score under 16 on the FAB scale, a threshold that suggests mild cognitive impairment in individuals with PD [54]. Finally, on the SCOPA-cognition scale, 41.7% of the sample scored below 24, a cutoff value indicative of cognitive impairment [55].

### 3.1. Reliability of Balance Outcomes During Single-Task Condition

Balance performance outcomes under the single-task condition are presented in Table 2. No statistically significant differences were observed across any of the outcomes measured between Test 1 and Test 2, as indicated by the paired tests (*p* > 0.05). Overall, test–retest reliability was excellent for most outcomes, including mediolateral CoP movement (ICC 0.83 to 0.89), CoP velocity (ICC = 0.90), Swept area (ICC = 0.92), and anteroposterior CoP dispersion and force (ICC = 0.79 and 0.90, respectively). Only anteroposterior CoP displacement showed fair-to-good reliability (ICC = 0.73). Notably, among the outcomes demonstrating excellent reliability, the precision of the point estimates differed. CoP swept area and mean CoP velocity exhibited the narrowest 95% confidence intervals, reflecting greater precision in their reliability estimates, whereas outcomes such as anteroposterior CoP dispersion presented comparatively wider confidence intervals.

Regarding absolute measurement error under single-task condition, SEM and MDC for each outcome are reported in Table 2, providing the specific thresholds required to distinguish true clinical change from measurement noise in original units.

### 3.2. Reliability of Balance Outcomes During Dual-Task Conditions

In the DT conditions, the registered variables showed lower reliability values than those in the single-task condition (Table 3 and Table 4). Firstly, the swept area did not prove reliable in the cognitive and DT_lower_ conditions, as the confidence intervals of the calculated ICCs contained 0 or were close to it. In contrast, this outcome showed excellent reliability in the DT_upper_ condition, with a confidence interval between 0.52 and 0.97. In this condition, a statistically significant difference was observed between Test 1 and Test 2; however, the median difference remained below the MDC despite exceeding the SEM (Table 4). On the other hand, CoP velocity proved to be the most reliable CoP parameter under DT conditions, as it yielded the highest ICCs with the narrowest confidence intervals, particularly in the DT_verbal_, where both the ICC and its 95% confidence interval demonstrated excellent reliability (Table 3). Although the paired differences for CoP velocity were significant across all DT conditions, the median differences remained below their respective SEM and MDC thresholds.

Meanwhile, mediolateral CoP displacement and dispersion showed greater reliability than those in the anteroposterior direction during the DT conditions. Under the cognitive tasks, mediolateral CoP displacement and dispersion all reached ICC values equal to or above 0.75 (Table 3), with a confidence interval whose lower bound is not less than 0.40 (indicating fair-to-good reliability). Furthermore, the difference between Test 1 and Test 2 was not statistically significant, with the median difference remaining below the calculated SEM and MDC values. In contrast, under both cognitive conditions, the confidence intervals for the anteroposterior displacement and dispersion parameters included or approached zero, reflecting poor reliability. Regarding force, anteroposterior CoP force demonstrated fair-to-good reliability and excellent reliability in the DT_visual_ (ICC = 0.75) and DT_verbal_ (ICC = 0.82) conditions, respectively, whereas the confidence intervals for mediolateral CoP force included values below 0.40 in both conditions.

The aforementioned pattern slightly changed under the motor conditions. Under the DT_upper_ condition, the mediolateral and anteroposterior parameters showed greater reliability than under the DT_lower_ condition. In the DT_upper_ condition, CoP dispersion and force in both directions, along with anteroposterior CoP displacement, reached ICC values above 0.75, demonstrating excellent reliability, with confidence intervals ranging from 0.48 to 0.96. However, under the DT_lower_ condition, the parameters with the best reliability were mediolateral dispersion and anteroposterior force, with an ICC greater than 0.80 and a confidence interval ranging from 0.49 to 0.92. Despite values indicating fair-to-good or higher reliability, it should be noted that only the anteroposterior CoP force showed a non-significant median difference below the calculated SEM value, establishing it as the most reliable parameter across the motor conditions.

### 3.3. Bland and Altman Plots for the Primary Outcome

**Figure 2:**
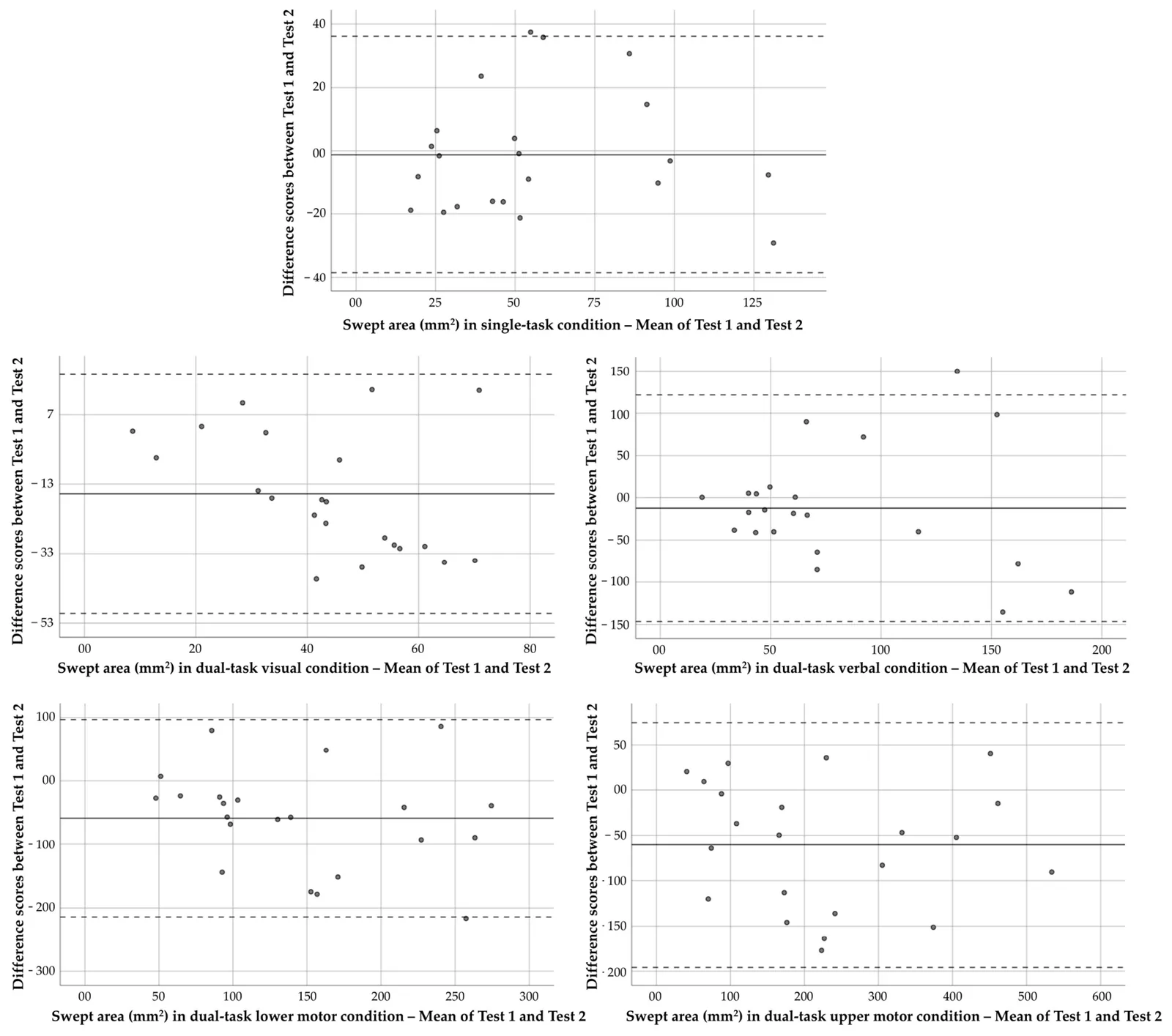
Bland and Altman plots for Swept area during single-task and dual-task conditions, visual, verbal, lower, and upper motor with the arms. The plots represent the difference between Test 1 and Test 2 (*Y*-axis) plotted against the Mean of Test 1 and Test 2 (*X*-axis). The average of the differences between Test 1 and Test 2 is presented as a continuous horizontal line on the plot. The upper and lower dashed lines represent the 95% upper and lower limits of the values plotted on the *Y*-axis.

For the primary outcome of this study, the results were further supported and displayed using Bland–Altman plots showing the means and differences between Test 1 and Test 2 (Figure 2). While the single-task condition showed relatively narrow limits of agreement (36.16 to −38.48), as did the DT_visual_ condition (18.61 to −50.21), the remaining DT conditions showed greater dispersion and substantially wider limits of agreement. Specifically, the limits expanded to 121.65 and −146.43 in the DT_verbal_ condition, 96.84 and −214.08 in the DT_lower_ condition, and 74.48 to −195.80 in the DT_upper_ condition. When interpreted in conjunction with the paired test results, these wide intervals and dispersion patterns indicate that systematic differences and increased measurement variability are present in the more demanding DT scenarios.

## 4. Discussion

This study analyzed the intra-rater test–retest reliability of biomechanical balance assessment under single and DT conditions in untrained individuals with PD. The protocol involved two cognitive and two motor tasks to evaluate postural stability and anticipatory postural control. To our knowledge, one previous study has included daily-life secondary tasks in balance assessment [16], but without mentioning the cognitive status of their participants. Although secondary tasks increase variability, they replicate real-life scenarios where indoor falls occur [56]. Furthermore, including individuals with and without cognitive alterations—a frequent non-motor symptom in PD [57,58]—enhances external validity and represents clinical reality.

Our findings identified which force platform outcomes during the Romberg test are most stable for intervention designs. In the single-task condition, all variables showed excellent reliability except anteroposterior CoP displacement, which exhibited a fair-to-good ICC. The swept area yielded the best results, making it the most reliable parameter for assessing single-task balance changes in mild to moderate PD, followed by the CoP velocity. These findings align with reports on healthy young adults [59,60] and older populations [60]. In addition, the paired comparative test performed in the single-task condition revealed no significant differences between Test 1 and Test 2, with the median differences remaining below the SEM value calculated for each variable. Consequently, this demonstrates high measurement stability and minimal systematic error in the absence of secondary tasks. Lastly, the MDC reported in this study during the single-task condition provides essential thresholds for assessing whether true change has occurred following a balance intervention. For swept area, the MDC involves a reduction of 26.79 mm^2^, and for CoP velocity, 0.39 cm/s.

Under DT conditions, balance reliability decreased, suggesting that dividing attention increases measurement variability. This was reflected not only by lower calculated ICC values compared to the single-task condition, but also by the confidence intervals of these parameters, which revealed lower limits below 0.40 and even close to zero for some variables. Performing a secondary task simultaneously with postural control requires the allocation of attentional resources between both activities, which may increase trial-to-trial fluctuations. Additionally, variations in secondary task performance and cognitive status among participants likely contributed to reduced reliability. For instance, the sway area was not a reliable variable under either the cognitive conditions or the DT_lower_, which can be explained by intermittent fluctuations in postural control due to divided attention, an increase in the intrinsic variability of participants with PD during the DT conditions, and the execution of a manual task off the body midline, thereby promoting an elevated CoP excursion area. Furthermore, because the swept area encompasses both mediolateral and anteroposterior movements, it is inherently more prone to fluctuations when facing secondary task demands. This finding is consistent with Terra et al., who reported an ICC close to poor reliability (ICC = 0.42; 95% CI = −0.46 to 0.77) for CoP area under a mathematical DT condition.

Conversely, CoP velocity showed the highest ICC with the narrowest confidence intervals across the DT conditions (ICC > 0.75 in DT_verbal_ and both motor conditions), suggesting that sway velocity is a more reliable outcome than total excursion area. The clinical importance of CoP velocity has been highlighted by its ability to distinguish PD fallers from non-fallers with a 0.57 cm/s difference [40]. However, these findings must be interpreted with caution, as the calculated SEM—and consequently the MDC values—for CoP velocity under DT conditions ranged from 0.39 to 0.71, exceeding those reported in previous studies using a mathematical DT [16]. Nonetheless, this measurement error could potentially be minimized with a larger sample size. Lastly, in our study, we observed significant differences in the paired tests for CoP velocity in the DT conditions. The presence of significant differences between Test 1 and Test 2, despite high reliability, underscores the distinction between relative consistency and absolute agreement, often reflecting minor learning or familiarization effects across sessions.

Furthermore, mediolateral variables were more reliable than anteroposterior outcomes across DT conditions, but particularly under the visual, verbal, and DT_lower_ conditions. This agrees with prior reports in PD under single-task conditions [16], and with CoP amplitude reliability in healthy adults during static and dynamic balance tests [59]. A potential explanation is that the larger footprint surface area available for anteroposterior sway may allow greater motion, which could contribute to smaller mediolateral fluctuations and lower inter-trial variability. Additionally, anteroposterior motion control during standing occurs mainly at the ankle, whereas mediolateral balance depends on the hip abductor/adductor muscles [61]. As in PD, rigidity and bradykinesia are more pronounced in distal joints [62,63]; therefore, anteroposterior control may be more variable. Finally, DT cognitive load might interfere more with anteroposterior postural control, which relies heavily on visual input [64]. Despite this, CoP force showed the opposite pattern, with higher reliability in the anteroposterior direction than in the mediolateral direction across DT conditions (ICC from 0.75 to 0.88), likely because it relates to body weight and displacement velocity. On the other hand, parameters appeared more reliable under the DT_upper_, likely because the manual task was performed precisely along the body midline, which may have promoted a more consistent postural sway compared to the motor task involving the arms positioned in front of the body.

### Study Limitations

Several limitations should be considered when interpreting these findings. The relatively small sample size may have affected the precision of the estimated ICCs, resulting in wider confidence intervals for certain parameters. Additionally, multiple comparisons (paired tests) were performed—given the number of variables and conditions included in the study—without adjusting the *p*-value for multiple comparisons. While this increases the likelihood of finding significant differences between Test 1 and Test 2 where none actually exist (Type I error), the general absence of significant differences across most parameters reinforces the stability of our findings and aligns with our high ICC values. However, the high number of comparisons and the statistical differences found could cause us to underestimate the stability of an outcome.

On the other hand, secondary task performance during DT was not formally quantified, limiting standardization and introducing additional variability, which prevents us from distinguishing it from the intrinsic fluctuations associated with PD. Moreover, the short 30-min rest interval between testing sessions may have been insufficient to completely wash out fatigue or familiarization effects, suggesting that longer intervals should be implemented in future short-term reliability studies. To minimize potential learning effects, the content of the cognitive tasks differed between Test 1 and Test 2, ensuring that participants did not encounter the same clocks or verbalize the same concepts in both assessments.

Furthermore, the study lacked healthy controls or a physiotherapy control group, preventing us from determining whether the observed variability was disease-related or inherent to the testing protocol. In addition, the heterogeneity of the sample regarding clinical characteristics, including the distribution of Hoehn and Yahr stages, the presence of previous falls, and cognitive status, may have contributed to variability in postural performance and influenced the reliability estimates. Finally, the single-center design limits the generalizability of our results.

## 5. Conclusions

This study found excellent short-term intra-rater reliability of single-task static balance measures in people with mild-to-moderate PD before a physical therapy intervention. Under single-task conditions, swept area appears to be the most reliable outcome to assess balance changes. Under DT conditions, CoP velocity and mediolateral outcomes showed relatively higher reliability than other outcomes, suggesting their potential utility as markers of postural control in PD. Nevertheless, limitations such as the lack of a control group comparison and secondary task monitoring should be acknowledged. Future research should investigate the responsiveness of these variables to balance physiotherapy interventions, their predictive validity for fall risk, and the influence of disease and cognitive status on reliability estimates.

## Figures and Tables

**Table 1:** Participants’ descriptive data.

Characteristics	Mean (SD)/Frequency	Median	Range/Percentage (%)
**Demographic & Anthropometric**			
Age (years)	65.82 (9.46)	70	40–76
Sex (Male/Female)	M: 14; F: 8		M: 63.6%; F: 36.4%
Height (m)	1.66 (0.10)	1.63	1.50–1.96
Weight (kg)	73.69 (15.30)	72.95	44.30–108.90
BMI	26.82 (4.27)	27.11	18.92–40.24
**Clinical & Neurological Status**			
PD evolution (years)	7.64 (4.93)	7	2–20
H&Y stages	I: 5; II: 8; III: 9		I: 22.7%; II: 36.4%; III: 40.9%
UPDRS III	19.36 (9.03)	16	6–41
LED (mg/day)	623.29 (339.31)	550.25	126–1650
**Cognitive Profile**			
MMP (_/32)	28.50 (3.55)	29	22–32
FAB (_/18)	15.83 (2.03)	17	12–18
SCOPA_Cog (_/43)	23.42 (6.21)	25.50	13–32

Abbreviations: MMP, Mini-Mental Parkinson Test. FAB, Frontal Assessment Battery. SCOPA_Cog, Scales for Outcomes in Parkinson’s disease-Cognition. BMI, body mass index. UPDRS III, Unified Parkinson’s Disease Rating Scale, part III (motor assessment). LED, Levodopa Equivalent Dosis. M, male. F, female.

**Table 2:** Intra-rater re-test Romberg test with eyes open in single-task.

Outcomes	Test 1	Test 2	Test 1 − Test 2	*p*-Value	ICC (95% CI)	SEM	MDC
**CoP displacement**							
ML displacement (mm)	15.92 (6.62)	14.39 (6.92) *	0.00	0.28	0.83 (0.59–0.93)	2.76	7.64
AP displacement (mm)	20.13 (5.68)	19.87 (7.28) *	0.65	0.37	0.73 (0.38–0.89)	3.24	8.98
**Global measures**							
Swept area (mm^2^)	56.30 (35.37)	49.39 (46.56) *	0.83	0.49	0.92 (0.81–0.96)	9.67	26.79
Velocity (cm/s)	0.98 (0.64) *	1.05 (0.59) *	0.07	0.37	0.90 (0.76–0.95)	0.14	0.39
**CoP dispersion**							
ML dispersion (mm)	2.88 (1.15) *	2.87 (2.98) *	0.01	0.66	0.86 (0.67–0.94)	0.50	1.39
AP dispersion (mm)	4.11 (1.40) *	4.11 (1.39)	0.21	0.41	0.79 (0.52–0.91)	0.66	1.83
**CoP Force**							
ML force (N)	8.41 (3.39)	8.48 (2.82)	0.07	0.86	0.89 (0.75–0.95)	1.00	2.76
AP force (N)	4.55 (2.95) *	5.46 (2.47)	0.38	0.16	0.90 (0.76–0.95)	0.71	1.97

Data are presented as mean and standard deviation (SD) for normally distributed variables, and as median and interquartile range for non-normally distributed outcomes (*). Differences between Test 1 and Test 2 were evaluated using paired-sample *t*-tests for normally distributed data and Wilcoxon signed-rank tests for non-normally distributed data. ML, mediolateral. AP, anteroposterior. *t*-test, value of *t*-student test. ICC, Intraclass Correlation Coefficient. CI, confidence interval. SEM, standard error of measurement. MDC, minimal detectable change.

**Table 3:** Intra-rater re-test Romberg test with eyes open in cognitive dual-tasks.

Outcomes	Test 1	Test 2	Test 1 − Test 2	*p*-Value	ICC (95% CI)	SEM	MDC
***Visual dual-task***							
**CoP displacement**							
ML displacement (mm)	15.68 (5.98)	14.34 (10.28) *	0.05	0.39	0.75 (0.41–0.89)	5.00	13.86
AP displacement (mm)	20.13 (5.68)	22.67 (8.98) *	1.91	0.22	0.54 (−0.04–0.80)	5.66	15.70
**Global Measures**							
Swept area (mm^2^)	34.11 (16.93) *	51.56 (22.14)	18.24	0.01	0.60 (−0.07–0.84)	11.96	33.16
Velocity (cm/s)	1.27 (0.61) *	1.11 (0.44) *	0.16	0.01	0.74 (0.36–0.89)	0.71	1.95
**CoP dispersion**							
ML dispersion (mm)	2.95 (1.44) *	2.83 (2.60) *	0.12	0.16	0.81 (0.55–0.92)	0.72	2.04
AP dispersion (mm)	4.06 (1.27)	4.72 (1.68)	0.66	0.02	0.74 (0.37–0.89)	0.75	2.07
**CoP Force**							
ML force (N)	8.43 (3.27)	9.13 (5.13) *	1.15	0.08	0.58 (0.05–0.82)	3.13	8.69
AP force (N)	5.28 (5.70) *	4.24 (5.65) *	1.04	0.20	0.75 (0.40–0.90)	2.33	6.46
***Verbal dual-task***							
**CoP displacement**							
ML displacement (mm)	19.14 (6.55)	17.21 (10.57) *	0.87	0.49	0.84 (0.62–0.93)	3.18	8.82
AP displacement (mm)	20.08 (6.57)	25.14 (11.85) *	3.35	0.00	0.60 (0.03–0.83)	5.66	15.68
**Global measures**							
Swept area (mm^2^)	53.46 (82.73) *	62.30 (58.52) *	8.84	0.22	0.50 (−0.19–0.79)	41.75	115.74
Velocity (cm/s)	1.70 (1.05) *	1.32 (0.78) *	0.38	0.02	0.92 (0.80–0.96)	0.39	1.08
**CoP dispersion**							
ML dispersion (mm)	3.82 (1.41)	3.67 (2.67) *	0.08	0.12	0.86 (0.66–0.94)	0.55	1.53
AP dispersion (mm)	4.32 (1.95)	5.40 (2.89) *	1.48	0.00	0.66 (−0.09–0.88)	1.03	2.86
**CoP Force**							
ML force (N)	9.32 (3.28)	11.63 (4.83)	2.31	<0.01	0.81 (0.28–0.93)	1.79	4.96
AP force (N)	7.38 (3.45)	8.11 (5.48)	0.73	0.34	0.82 (0.58–0.92)	1.91	5.28

Data are presented as mean and standard deviation (SD) for normally distributed variables, and as median and interquartile range for non-normally distributed outcomes (*). Differences between Test 1 and Test 2 were evaluated using paired-sample *t*-tests for normally distributed data and Wilcoxon signed-rank tests for non-normally distributed data. ML, mediolateral. AP, anteroposterior. *t*-test, value of *t*-student test. ICC, Intraclass Correlation Coefficient. CI, confidence interval. SEM, standard error of measurement. MDC, minimal detectable change.

**Table 4:** Intra-rater re-test Romberg test with eyes open in motor dual-tasks.

Outcomes	Test 1	Test 2	Test 1 − Test 2	*p*-Value	ICC (95% CI)	SEM	MDC
***Lower motor dual-task: below 90° of shoulder flexion***							
**CoP displacement**							
ML displacement (mm)	27.28 (11.59) *	32.40 (11.47)	3.55	0.04	0.74 (0.39–0.89)	6.56	18.19
AP displacement (mm)	24.71 (5.07)	29.87 (7.26)	5.16	<0.01	0.48 (−0.12–0.78)	4.48	12.43
**Global measures**							
Swept area (mm^2^)	91.61 (117.30) *	175.43 (90.11)	70.94	0.01	0.60 (0.01–0.83)	51.73	143.38
Velocity (cm/s)	1.73 (1.24) *	1.71 (1.36) *	0.02	0.07	0.79 (0.48–0.91)	0.60	1.66
**CoP dispersion**							
ML dispersion (mm)	5.92 (3.01) *	7.15 (2.66)	0.76	0.01	0.81 (0.49–0.92)	1.09	3.02
AP dispersion (mm)	4.74 (1.11)	6.29 (1.77)	1.55	<0.01	0.48 (−0.19–0.78)	1.07	2.95
**CoP Force**							
ML force (N)	9.70 (4.59) *	13.25 (4.50)	3.30	0.00	0.79 (0.27–0.92)	2.00	5.55
AP force (N)	6.51 (4.71) *	7.19 (4.83) *	0.68	0.72	0.80 (0.52–0.91)	1.55	4.31
***Upper motor dual-task: above 90° of shoulder flexion***							
**CoP displacement**							
ML displacement (mm)	30.79 (9.62)	40.01 (15.17)	9.22	<0.01	0.73 (0.01–0.91)	4.70	13.03
AP displacement (mm)	33.64 (9.59)	38.07 (12.03)	4.43	0.02	0.80 (0.48–0.92)	4.87	13.49
**Global measures**							
Swept area (mm^2^)	142.89 (212.23) *	258.02 (152.76)	96.32	0.00	0.90 (0.52–0.97)	46.20	128.07
Velocity (cm/s)	2.22 (1.67) *	2.30 (0.98) *	0.08	0.06	0.83 (0.58–0.92)	0.69	1.93
**CoP dispersion**							
ML dispersion (mm)	6.46 (2.22)	7.46 (2.45)	1.00	<0.01	0.89 (0.49–0.96)	0.75	2.08
AP dispersion (mm)	6.74 (2.39)	7.71 (4.43)	0.97	0.02	0.77 (0.44–0.91)	1.14	3.15
**CoP Force**							
ML force (N)	14.75 (5.63)	17.10 (7.39)	2.35	0.02	0.84 (0.58–0.94)	2.61	7.22
AP force (N)	10.84 (4.58)	11.18 (8.06) *	1.38	0.18	0.88 (0.72–0.95)	1.98	5.49

Data are presented as mean and standard deviation (SD) for normally distributed variables, and as median and interquartile range for non-normally distributed outcomes (*). Differences between Test 1 and Test 2 were evaluated using paired-sample *t*-tests for normally distributed data and Wilcoxon signed-rank tests for non-normally distributed data. ML, mediolateral. AP, anteroposterior. *t*-test, value of *t*-student test. ICC, Intraclass Correlation Coefficient. CI, confidence interval. SEM, standard error of measurement. MDC, minimal detectable change.

## Data Availability

The datasets used and analyzed during the present study can be requested from the corresponding author.

## Abbreviations

The following abbreviations are used in this manuscript:

DT	Dual-task
PD	Parkinson’s disease
SEM	Standard Error of Measurement
MDC	Minimal Detectable Change
ICC	Intraclass Correlation Coefficient
CoP	Center of Pressure
DT_visual_	Visual dual-task
DT_verbal_	Verbal dual-task
DT_lower_	Lower motor dual-task performed with the arms positioned below 90° shoulder flexion
DT_upper_	Upper motor dual-task performed with the arms raised above 90° shoulder flexion
